# Analysing Interlinked Frequency Dynamics of the Urban Acoustic Environment

**DOI:** 10.3390/ijerph192215014

**Published:** 2022-11-15

**Authors:** Timo Haselhoff, Tobias Braun, Jonas Hornberg, Bryce T. Lawrence, Salman Ahmed, Dietwald Gruehn, Susanne Moebus

**Affiliations:** 1 Institute for Urban Public Health (InUPH), University Hospital Essen, University of Duisburg-Essen, 45147 Essen, Germany; 2 Complexity Science, Potsdam Institute for Climate Impact Research, 14473 Potsdam, Germany; 3 Department of Spatial Planning, TU Dortmund University, 44227 Dortmund, Germany

**Keywords:** urban soundscape, acoustic environment, frequency correlation matrices, time–frequency domain, urban acoustics

## Abstract

As sustainable metropolitan regions require more densely built-up areas, a comprehensive understanding of the urban acoustic environment (AE) is needed. However, comprehensive datasets of the urban AE and well-established research methods for the AE are scarce. Datasets of audio recordings tend to be large and require a lot of storage space as well as computationally expensive analyses. Thus, knowledge about the long-term urban AE is limited. In recent years, however, these limitations have been steadily overcome, allowing a more comprehensive analysis of the urban AE. In this respect, the objective of this work is to contribute to a better understanding of the time–frequency domain of the urban AE, analysing automatic audio recordings from nine urban settings over ten months. We compute median power spectra as well as normalised spectrograms for all settings. Additionally, we demonstrate the use of frequency correlation matrices (FCMs) as a novel approach to access large audio datasets. Our results show site-dependent patterns in frequency dynamics. Normalised spectrograms reveal that frequency bins with low power hold relevant information and that the AE changes considerably over a year. We demonstrate that this information can be captured by using FCMs, which also unravel communities of interlinked frequency dynamics for all settings.

## 1. Introduction

The WHO defines the effects of the acoustic environment (AE) in the form of noise as “one of the most important environmental risks to health” [1]. Accordingly, the impact of noise in urban regions is well-researched [2,3,4,5,6,7,8,9], while less is known about the health effects of the (urban) AE above and beyond noise [10]. Low-frequency sound as well as infrasound have long been thought to impact human health [11,12,13], but solid evidence is still lacking. More recently, different approaches (e.g., perceptual soundscape research) are being used to study the impact of the audible AE on human health and well-being [14]. Here, first results show associations between the perceptions of the AE and, for example, induced stress or recovery periods from (psychological) stress [15,16,17]. Still, the assessment of the AE on a larger scale is limited to sound pressure levels, and the (audible) time–frequency domain of the urban AE is only rarely assessed [18,19].

In contrast, the time–frequency domain plays an important role in other research fields, and methods are established to retrieve more information of the AE than just sound pressure levels. In ecoacoustics, the AE is monitored to access information about biodiversity or species abundance, or to study the impacts of climate change [20,21,22,23,24,25]. Here, spectral information is used to calculate indices that measure the complexity, diversity or entropy of the AE. Unfortunately, such methods are not readily transferable, as assumptions made for ecoacoustic indices and sound sources do not always hold true and are difficult to evaluate in the urban environment [26,27,28]. For instance, Bradfer-Lawrence et al. state that for ecoacoustic indices “there are often competing explanations for a particular index” [26], and Fairbrass et al. show that biasing sounds (e.g., road traffic) must be removed for an appropriate use of ecoacoustic indices in the urban environment [27], a task, that is—at the time—impossible to accomplish with data that comprise thousands of hours of audio recordings.

An additional reason for the difficulty in evaluating the urban AE is that data on longitudinal measurements of the audible spectrum are scarce, as systematically sampled, high-quality audio recordings are expensive in planning, execution, equipment, computational power and storage space [29]. Thus, knowledge about the temporal and spatial frequency dynamics of the urban AE is lacking, and no general guidelines on how to assess the audible spectrum of the urban AE exist. However, in recent years, the decreased cost of acoustic sensors, storage space and computational power has led to a rapid increase in the popularity of passive acoustic monitoring (PAM) [29]. Therefore, longitudinal studies of the AE from multiple urban settings—recording the whole audible frequency spectrum—have become achievable. One example of such a study is SALVE (AcouStic QuAlity and HeaLth in Urban EnVironmEnts). Briefly, SALVE is a longitudinal study on the acoustic quality of urban spaces in Bochum, Germany [30]. This enables analyses of higher temporal and spatial resolution data of the urban AE.

Accordingly, the main goal of this work is to support evidence for and to provide a better understanding of the spectral power distribution as well as frequency dynamics of the urban AE. For this, we use SALVE data from nine selected locations over 271 days to analyse the urban AE across the audible frequency spectrum. We study the spectral content and frequency dynamics of the recordings using averaged power spectra as well as normalised spectrograms for all nine settings. Another problem in environmental acoustics exhibits from the visualisation and analysis of long period time–frequency data, as typical spectrograms (e.g., temporal scale of 0.02 s/frame and one frame per pixel column) cannot be displayed on standard monitors, even for only one 24 h recording [31]. Therefore, we additionally propose a method using frequency correlation matrices (FCMs) to analyse the urban AE, by capturing links between the variability in different frequency bands. Correlation matrices are a common tool to describe associations between a plethora of variables and build the foundation, e.g., for connecting cortical hubs in the human brain [32] or climate dynamics [33]. Unlike more commonly used methods, such as spectrogram cross-correlation [24], FCMs have been only seldom used in the field of acoustics, e.g., to describe ocean ambient noise [34,35]. In the urban acoustic environment, high correlations between frequency bands characterise the prevalence of particular sound sources and therefore provide valuable information to distinguish between the AEs of different urban settings. In contrast to many existing methods, FCMs consider the entire time–frequency domain and yield a rich representation of the frequency dynamics of the urban AE. In addition, the dimension of FCMs is only dependent on the number of frequency bands. Thus, there are virtually no restrictions on the selection of the number of audio recordings to be analysed and visualised.

In summary, the objective of our work is to contribute to a better understanding of frequency dynamics in the urban AE and to provide a versatile method to analyse and represent large datasets of audio recordings from the urban AE, exploiting as much information from the time–frequency domain as possible. In this way, we enable a better understanding and interpretation of the urban acoustic environment, characterising frequency dynamics in addition to the limited information from sound pressure levels alone. This will help to improve future research such as perception-based and psychoacoustic approaches by quantifying additional properties of the (audible) time–frequency domain to understand its impact on human health.

## 2. Data

For our analyses, we used data from the SALVE study. SALVE is a cooperative project of the Institute for Urban Public Health (InUPH) of the University of Duisburg-Essen and the Research Group Landscape Ecology and Landscape Planning (LLP) at TU Dortmund University. Briefly, 50 audio recordings each day have been recorded at more than 50 locations in Bochum since 2019. Meanwhile, data are available from over a period of more than three years. For this work, we analysed a subset from nine different locations, representing nine different built environments. The recordings were made from May 2019 to the end of February 2020. The choice of endpoint was motivated by the COVID-19 pandemic and the resulting drastic changes in the acoustic environment [36,37,38].

In total, the analysed dataset consists of 130,017 3-min recordings made at nine different locations, from 7 May 2019 to 25 February 2020. In total, this equals 390,051 min or 271 days of consecutive audio recordings. To the best of the authors’ knowledge, this makes it one of the most comprehensive urban AE datasets analysed to date.

### 2.1. Recording Methods

All recordings were made by Wildlife Acoustics SM4 recorders with a SMMA2 microphone [39]. The devices were mounted at a height of approx. 1.65 m [40] and programmed to record 3-min recordings every 26 min to optimise battery performance and reduce data storage. Following DIN ISO 12913-1:2018-02 [40], the sampling frequency was set to 44.1 kHz, which corresponds to the human hearing range of about 20 kHz. The bit depth was set to 16, since this is the maximum possible bit depth for SM4 recorders.

### 2.2. Land Use Types

**Figure 1:**
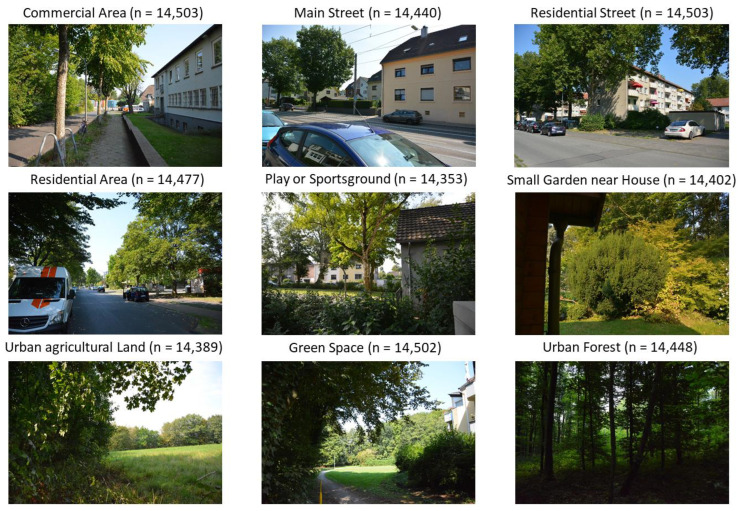
Pictures and classification of the urban environment around all nine recording devices. The number in brackets is the number of 3-min recordings per site. In total, 130,017 recordings were used for this work.

The initial definition of the built environment was based on the land use types (LUTs) provided by the Ruhr Regional Association [41]. As the recorders were sometimes placed close to the edges of the land use polygons, we defined categories that reflected the actual urban environment more appropriately. For this, we considered the original LUTs, photographs, and assessments of the respective recording sites. Following this, each team member carried out the classification separately. Disagreements were solved through discussion between the team members. The resulting LUTs and respective pictures of the recording sites can be found in Figure 1.

**Figure A1:**
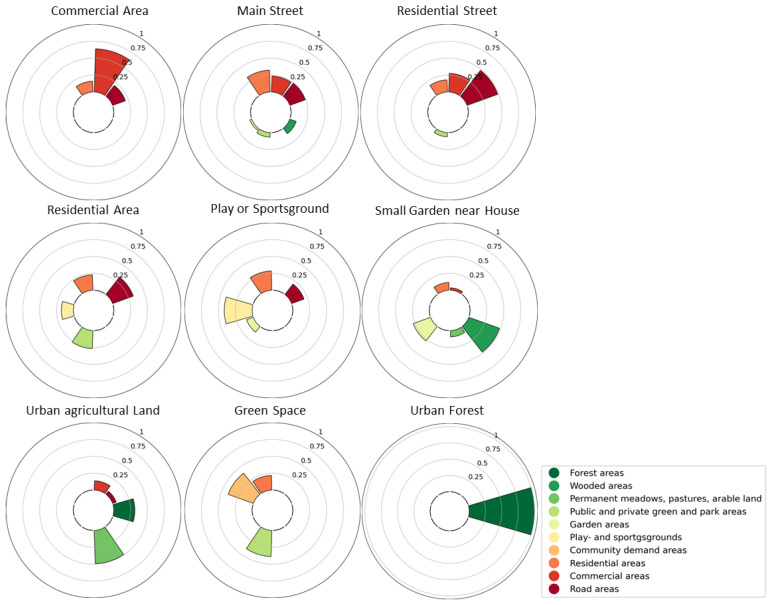
Percentage of land use area (as defined by Ruhr Regional Association [41]) in a 50 m radius buffer around each recording station. Depicted are the ten largest land use types, which in total occupy more than 94% of the 50 m buffer area.

The order of the representation in Figure 1 follows a gradient from urban areas to areas that are more natural. The land use mix (LUM)—as defined by the Ruhr Regional Association [41]—around all recording stations can be found in Appendix A (Figure A1). It shows that the surroundings of “Commercial Area”, “Main Street” and “Residential Street” are dominated almost purely by road, commercial and residential areas. “Residential Area” and “Play- or Sportsground” also include garden areas as well as public and private green spaces. The LUM of the other four recording stations are characterised mainly by natural areas like forest and wooded areas. Only for “Green Space”, the environment includes again more built-up areas.

## 3. Materials and Methods

This section is divided by subheadings. It provides a concise and precise description of the materials and methods, their interpretation, as well as the conclusions that can be drawn.

### 3.1. Pre-Processing

Pre-processing consisted of three steps: (1) plausibility check of the data, (2) calculation of the power spectra for each recording and (3) de-noising and outlier removal. As we wanted to ensure robustness of the results for all pre-processing steps, we considered as many data as possible. Thus, pre-processing was performed using the entire dataset from SALVE, defined in Haselhoff et al. [30] as an automatic aural procedure (AAP_24_). Briefly, the AAP_24_ dataset comprises 416,797 3-min recordings from 23 LUTs.

#### 3.1.1. Plausibility Check

The detailed description of the plausibility check for SALVE can be found in Haselhoff et al. [30]. To exclude faulty recordings or recordings with device-induced sounds (e.g., rattling of the device in extreme wind), a number of ecoacoustic indices (NDSI, BIO, ADI, AEI, ACI, Hf, Ht, H) [24] as well as sound pressure indices (LAeq_min, LAeq_max, LAeq_mean) were calculated for all recordings of the SALVE study. Subsequently, all indices were examined for abnormalities by means of descriptive statistics to exclude erroneous recordings.

#### 3.1.2. Calculation of Power Spectra

To the best of the knowledge of the authors, literature regarding signal processing of the urban acoustic environment lacks research defining optimal frequency bin sizes for summarising the signal in the frequency domain. Binning is necessary to a safe storage place, i.e., to reduce the dimensionality of the dataset for further analysis. Widely used R packages to calculate ecoacoustic indices such as seewave [42] or sound-ecology [43] use a window length of 512 as the default setting but no reason for that is given, neither in the documentation nor in the original works for each index.

To obtain an adequate binning of power spectra, we seek to identify a bin count that is high enough to conserve the complexity of spectra but simultaneously entails a significant reduction in the number of bins. To deduce this number, we take a simple random sample from all SALVE recordings, assuming a margin of error of 1% and a confidence level of 99%, which results in a sample size of 16,000 recordings [44]. Then, we calculate the power spectra for all recordings using Fast Fourier Transform (FFT) [45] and bin the signal, using 50 different bin counts B from 50 to 4000 bins with equal size. We quantify the complexity of spectra by two measures: (1) intra-bin variance and (2) spectral entropy. Based on these two measures, a binned power spectrum is considered complex if it exhibits a broad diversity of averaged spectral power over all frequency bins and, within each bin, does not eliminate too much variability. An appropriate binning should conserve as much as possible of the spectral complexity with a minimum number of bins.

For all 50 bin counts of each recording, intra-bin variance is calculated and subsequently averaged over all bins for the respective bin count. This results in 50 averaged intra-bin variance values for each recording. These values are then averaged by bin count over all 16,000 recordings to summarise the statistic for the whole sample:

(1) $$\bar{v}\left(B\right)=\frac{1}{n}\overset{n}{\underset{i=1}{\sum }}\frac{1}{B}\overset{B}{\underset{b=1}{\sum }}var\left(s_{ib}\right)$$

where $\bar{v}\left(B\right)$ is the mean intra-bin variance given a binning with B bins, $s_{ib}$ is the Fourier transform signal of the ith recording inside the bth bin and n is the total number of recordings. $Var\left(s_{ib}\right)$ is the variance of all spectral power values in the bth bin of $s_{i}$.

Spectral entropy is derived by calculating the Shannon evenness [46] of each recording’s power spectrum and averaging bin-wise for 50 different binnings:

(2) $$\bar{H}\left(B\right)=-\frac{1}{n}\overset{n}{\underset{i=1}{\sum }}\bar{s_{i}}\left(B\right)ln\bar{s_{i}}\left(B\right)\;,\;\;with\;\;\;\;\;\;\;\;\;\bar{s_{i}}\left(B\right)=\frac{1}{B}\overset{B}{\underset{b=1}{\sum }}s_{ib}$$

where $\bar{H}\left(B\right)$ is the mean spectral entropy given a binning with B bins,$\;\bar{s_{i}}\left(B\right)$ is the average of all values in the bth bin of $s_{i}$ and n is the total number of recordings.

**Figure 2:**
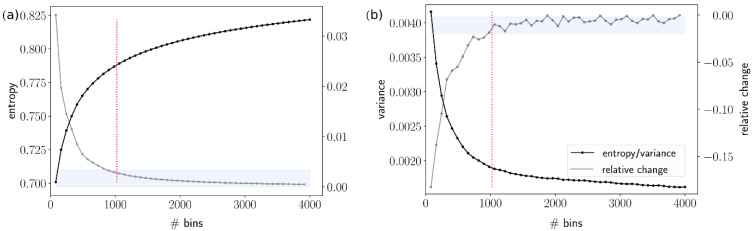
Spectral entropy (**a**) and intra-bin variance (**b**) for 50 different bin counts, averaged over 16,000 recordings (black line). The grey line displays the relative change of subsequent values to each other, and the blue shaded area indicates where this value is below 10% of its maximum change.

The aim here is to identify, for which bin size the intra-bin variance/spectral entropy does not drastically decrease/increase anymore, indicating separation precision between frequency bins. Results for the calculation of the power spectra to determine a feasible bin count can be found in Figure 2. If the number of bins is increased progressively, it shows that the relative change in both measures saturates for decreasing bin sizes. As indicated by the blue shading, both measures cease to change by more than 10% of their maximum change for a number of bins that exceeds approx. 1000 bins. Following common bit-conventions, we conclude that 1024 bins are sufficient for our recordings of the urban AE. Accordingly, we calculate the FFT for all recordings of our dataset, bin the values in 1024 equally sized bins and average the values inside each bin. The resulting time series represent temporal changes of spectral power for each frequency bin. As the magnitude of variability can broadly differ between low and high frequencies, we align them by a log transformation.

#### 3.1.3. De-Noising and Outlier Removal

We apply Principal Component Analysis (PCA) to reduce noise intensity in time evolution of each frequency band. PCA separates a dataset into orthogonal components from which each represents a linear combination of the original data. Ordered by their eigenvalues, the components explaining the least variance can be identified [47,48]. PCA is applied separately to each frequency bin for each LUT. We extract the leading principal components that describe ≥95% of the variance. From these, we reconstruct the corresponding part of the signal where each principal component is weighted by its respective eigenvalue. Therefore, approx. 5% of the explained variance of the original data is considered as noise. The resulting reconstructed signals still contain rare but strong excursions from their mean, which are likely due to isolated, loud events in the urban AE. We remove all values above the 99.95 Quantile for each frequency bin per location. The applied de-noising procedure and outlier removal enhance robustness of the following analyses [49].

### 3.2. Median Power Spectrum

To summarise the power spectra for all recordings per LUT, we calculate the median over all binned power spectra recordings, as it is more robust against large outlier than the mean. To still visualise the range of the power over all recordings we give the interquantile range (IQR) between the 5% and 95% quantile. Overall, this method provides an overview of the power distribution and its range for different frequencies in the urban AE.

### 3.3. Normalised Spectrograms

In order to analyse the evolution of frequency power over the span of ten months, we average the binned power spectra of all 50 recordings per location and per date. Subsequently, for each frequency (i.e., time series), the power is normalised separately for each frequency by its maximum power. Thus, a normalised power of one denotes the 10-month maximum value and zero the minimum measured power of the respective frequency bin. Consequently, the absolute power is not shown. The normalisation allows the representation of specific patterns in higher frequency bins, which would hardly be recognisable in the representation of the absolute power. This way, simultaneously occupied frequency bins are visualised. As temporal connections between frequency bins can be interpreted as similar sound sources [35] (s. 4.2), this method allows the characterisation of urban AEs by identifying interrelationships between frequency bins.

### 3.4. Correlation Matrices

**Figure A2:**
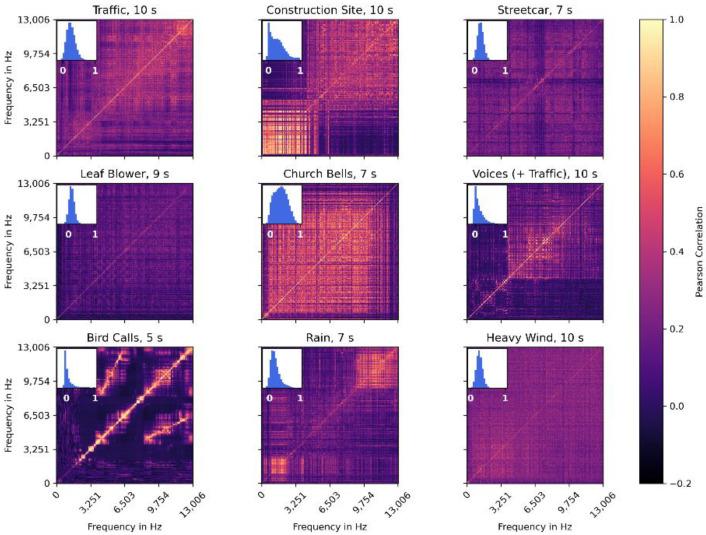
Sound source examples and their corresponding correlation matrices. A spectrogram was calculated for the corresponding audio recording. Then, power of all frequency bins were correlated over time for the period in which the sound source occurred. The Z-axis represents the Pearson correlation coefficient. The insert shows the distribution of all correlation values of the respective correlation matrix.

To characterise AEs by their frequency interrelationships, we calculate correlations between all frequency bins over time using Pearson’s correlation coefficient [50]. We calculate FCMs for all locations separately, while considering the entire recording period for each location. We report R^2^ as a measure of the proportion of explained variance between two frequencies. Here, high values of R^2^ indicate a strong relationship between frequencies, which is due to sound sources that occur simultaneously or closely subsequent in time and occupy multiple frequency bins. As occurrences of most sound sources are independent from each other (e.g., dogs barking, cars passing by, etc.), this method yields the opportunity to characterise different LUTs in regard to how their sound sources differ in prevalence from each other. For a more intuitive understanding of how FCMs work for specific sound sources, we show nine common sound source examples and their corresponding FCMs in Appendix A (Figure A2). Still, it is important to note that we correlate power spectra of 3-min recordings, therefore a high R^2^ between a pair of frequencies implies either the presence of a single sound source that occupies these two frequencies over several recordings (i.e., h) or multiple distinct sound sources that are linked in their occurrence over this time scale (3-min) (e.g., wind and rustling leaves). For this reason, and because the urban acoustic environment is always a mixture of a variety of sounds, FCMs are not intended to identify sound sources, but rather are used as a tool to characterise the particular LUT through its overall frequency dynamics over time.

In addition to the FCM, we report the histogram for the R^2^ values for each LUT, allowing us to analyse the distribution of correlation coefficients and therefore to identify multimodal distributions. Multimodal distributions can be seen as a mixture of multiple underlying distributions, potentially representing different groups [51]. In our case, a multimodal distribution results from the prevalence of different R^2^ values (i.e., correlated frequency bins) and may be an indicator for the diversity of sound sources.

## 4. Results

### 4.1. Median Power Spectrum

**Figure 3:**
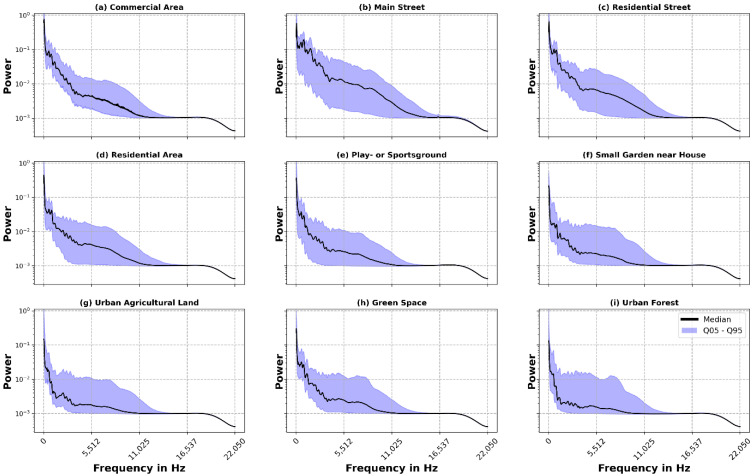
Log-scale frequency spectrum for all recordings, grouped by land use type (**a**–**i**). The black line represents the median of all recordings, and the blue area the range between the 5% and 95% quantile.

The Median Power Spectra in Figure 3 represent the distribution of power between frequency bins in the urban AE. The first feature that stands out as a commonality between all LUTs is a prominent peak of power in the frequency ranges from 0 up to about 80 Hz. This shows that a large share of power in the urban AE originates from sound in this frequency range. Ascending the frequency scale, the power drops remarkably but remains stable between 200 and 600 Hz, after which it drops again. In addition, a higher fluctuation of the median between consecutive frequency bins from 100 Hz to about 4.5 kHz can be observed, after which it becomes noticeably smoother. This shows a high diversity of power between consecutive frequency bins—likely from a variety of sound sources occupying different frequency bins in this range. Finally, we observe that the power in all land-use types diminishes above approx. 13 kHz and shows low variance, which can be seen by the very narrow IQR. Thus, the vast majority of sound sources in the urban environment occupy frequencies up to a maximum of about 13 kHz. Following this commonality between all LUTs, we limit following analyses to a range between 0 and 13 kHz. Regarding the IQR, another similarity between the LUTs is the relatively steady IQR for the frequency bins from 3 to 8 kHz. This indicates different sound sources, which, despite occupying different frequency ranges, have a similar distribution in power. In addition, clear differences can be seen between the LUTs with respect to the IQR. In particular, the 5% quantile for the locations represented by Figure 3a “Commercial Area”, Figure 3b “Main Street” and Figure 3c “Residential Street” does not exhibit a decline in power up to approx. 3 kHz as strong as in all other locations. This illustrates the dominance of the lower frequencies in these LUTs, which are presumably due to road traffic. Additionally, the median for the LUTs Figure 3a–e lies approx. in the middle of the IQR, while, for the other LUTs, it is located more in the lower half of the IQR. This speaks for more/fewer louder sounds in the first and latter LUTs, respectively.

### 4.2. Normalised Spectrograms

**Figure 4:**
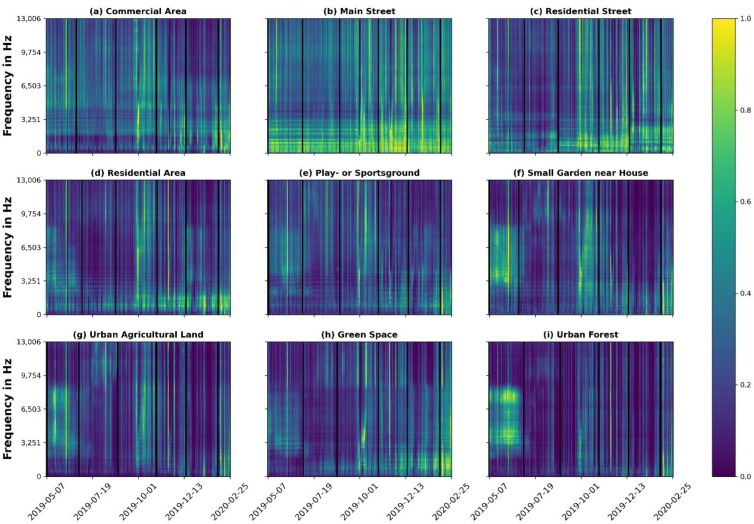
Normalised Spectrograms for all LUTs (**a**–**i**). A mean power spectrum was calculated for all recordings per date/device. Subsequently, for each frequency bin (i.e., time series), the power was normalised so that the values correspond to the relative power of the frequency to itself. Thus, one is the maximum measured power and zero is the minimum measured power of the respective frequency bin. Black columns represent maintenance days, during which no recordings were made.

Normalised Spectrograms reveal a plethora of information regarding normalised frequency power over time (Figure 4), from which we can only describe the most protruding results. Overall, we recognise a diversity of relative power between different frequency ranges and time periods. In comparison to all other LUTs, the power in all frequencies of Figure 4b “Main Street” does not seem to differ remarkably over the observation period of ten months. In addition, we see a prominent repeating pattern, especially in the low frequency range of the LUT “Main Street”, up to about 2.5 kHz. Here, longer sections of similar power are interrupted in regular intervals by short sections of lower power. Investigating the time series, it is noticeable here that the lower frequency powers are repeated in a weekly rhythm (i.e., on Saturday and Sunday). It can be assumed that this pattern reflects road traffic, which decreases noticeably on the weekend. Compared with the other examples, it is also noticeable that this pattern can be found—albeit more weakly—in almost all land use types and in ranges above 2.5 kHz. “Urban forest” alone does not reveal this pattern, which may represent an absence of traffic noise or anthrophonic sound sources in general.

Another pronounced pattern is found in the frequency range between 2.5 and 8.5 kHz. Here, high relative power can be seen from May to June for the LUTs Figure 4d) “Residential Area” and Figure 4e) “Play—or Sportsground, for which the latter shows the most prominent pattern. Following the assumption that bird sounds occur primarily in this frequency range [52] and with knowledge of the breeding season, this activity most likely reflects biophonic activity, i.e., bird songs. Another biophonic activity is present in the summer between approx. 9 and 11 kHz for Figure 4f,g,i. Investigating the time series once more, we find that these sounds occur in the evening hours and represent crickets. Regarding only relative power, we also see periods that seem to be ‘louder’ than others, independent from the frequency range. Here, the high relative power observed in October is noticeable and can be observed in almost all LUTs. One possible explanation would be strong winds, since they occupy the named frequency ranges and are more likely to be stronger in the fall. However, the exact reason for this pattern is unknown. This is most likely true for many other patterns that can be inferred from this figure and shows that the frequency dynamics of the urban AE are still not very well understood.

Nevertheless, by plotting the frequency power using a Normalised Spectrogram over the entire observation period, nontrivial patterns can be identified, which vary in time as well as in space and therefore show the high heterogeneity of the urban AE. Most prominent is the connection of different frequency ranges over time. It is reasonable to assume that temporally highly correlated frequency ranges are due to similar or the same sound sources (e.g., bird songs or car traffic). Consequently, a consideration of these correlations could offer a high potential for the differentiation and classification of different urban AEs, even if not all of them can be associated with a distinct source.

### 4.3. Correlation Matrices

**Figure 5:**
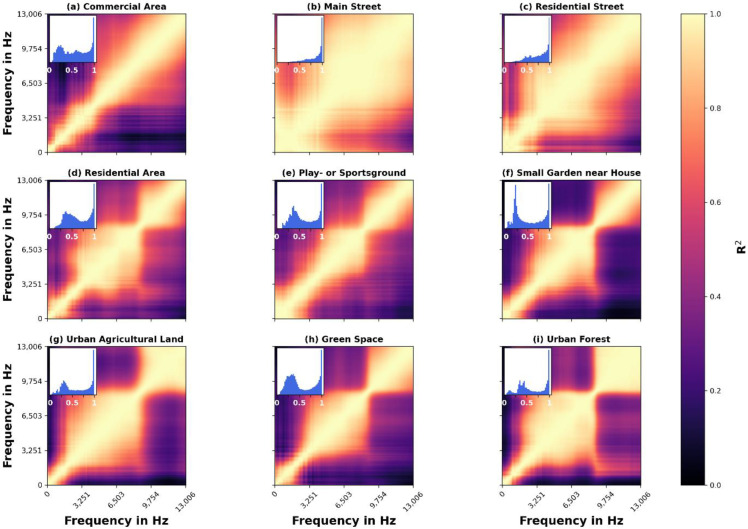
Frequency correlation matrices (FCMs) for all recordings over time, grouped by LUT (**a**–**i**). Here, the power spectra of each recording were correlated over time to identify temporally related frequency patterns. The Z-axis represents the squared Pearson correlation coefficient R^2^. The insert shows the distribution of all R^2^ values of the respective correlation matrix (diagonal 1s caused by correlating the frequency bin with itself were removed for this purpose).

As a result of the FCMs (Figure 5), clear rectangular group structures can be recognised in all LUTs, which represent distinct, correlated frequency power over time. First off, it is evident that clear differences in the frequency dynamics between all LUTs can be observed, while some of the matrices are more alike than others. We find that results from the Normalised Spectrograms are also captured by FCM. LUTs with prominent traffic noise (Figure 5b,c) show very distinct patterns. Here, we see that low frequencies < 100 Hz correlate highly with frequencies up to 9 kHz. Frequencies > 4 kHz also correlate strongly with almost all frequencies above. This suggests that only a few factors explain the overall variance of the acoustic environment, likely represented by road traffic. Histograms (inset) underline this impression, as the correlation values are close to unimodality. While the histogram of “Residential Street” still shows some small peaks around R^2^ = 0.5, indicating somewhat prominent additional sound interrelations, the overwhelming majority of values is close to 1. In contrast, LUTs for Figure 5d–i show clear differences in their FCMs and histograms. The striking commonality of these LUTs are the distinct square patterns. Here, frequencies > ~9.5 kHz consistently form a community. This finding is not obvious when looking only at the spectrograms from Figure 4. From there, one might be misled into assuming that this frequency range is connected to lower frequencies (<2.5 kHz) as in Figure 4b or Figure 4c. However, from correlation matrices we can derive that there are independent sound sources (in)active in this range. Only Figure 5d,e,h show higher correlations between >9.5 kHz and <700 Hz that can also be seen in the traffic-related LUTs. Although Figure 5d,i also show moderate-to-high correlations between >9.5 kHz and lower Hz (1–1.5 kHz), the latter range is different from the one observed in the “Main/Residential Street”.

Below 9.5 kHz, another square community is recognisable, but its lower frequency limit is more distinct between the LUTs. The range goes from approx. 2.5 kHz (“Urban Agricultural Land”) to approx. 4.5 kHz (“Play—or Sportsground”). Most likely, this pattern originates from the formerly mentioned bird songs in this frequency range. The differing lower bounds may be due to other sound sources active in-between these frequency ranges. Below the second square structure, frequency power no longer forms large coherent structures. A great diversity of sound sources likely exists in this frequency range, which is already reflected by the median power spectra (Figure 3). Following this assumption, fewer coherent structures are expected, as multiple sound sources may occupy different frequencies and occur at different time intervals. Consequently, these results underline the assumption that sound sources in the urban AE are more diverse up to a frequency range of about 4.5 kHz.

In addition, histograms indicate a higher diversity of prevalent sound sources in Figure 5d–i than in Figure 5b,c. LUTs from Figure 5d–i show similar patterns in R^2^-value distribution, but they differ in magnitude. All six LUTs show a multimodal distribution of R^2^ values, most of them with a second peak at around R^2^ = 0.4. While “Residential Area”, “Play- or Sportsground” and “Green Space” seem to be very similar, “Small Garden near house” shows a prevalence of R^2^ values close to 0.3 similar to those close to 1 (note that the diagonal ones that come from correlating the frequency bin with itself were removed).

Additionally, the histogram of Figure 5i “Urban Forest” shows a multimodal distribution with about three to four peaks. Both histograms indicate a higher prevalence of lesser-correlated frequency bins and, thus, more diverse sound sources.

## 5. Discussion

In this work, we described the urban acoustic environment using a spatially and temporally highly resolved dataset. To this extent, we gave a detailed description of pre-processing steps to ensure robustness of results. Using intra-bin variance and spectral entropy as evaluation metrics, we concluded that 1024 bins were sufficient to represent the urban AE on a linear frequency scale for the studied dataset. Investigation of the Median Power Spectra by LUT revealed that a majority of frequency power was located below 80 Hz and a high variance in power between frequency bins was observed up to about 4.5 kHz, followed by lower variance up to 13 kHz. Above 13 kHz, frequency bins were occupied only with very low, if any, power. However, clear differences in power and variance—especially in the range up to 9 kHz—were evident between LUTs. The examination of Normalised Spectrograms revealed prominent seasonal patterns in all frequency ranges. In addition, frequency groups with similar temporal dynamics were identified, some of which could be attributed to specific sound sources such as birds, crickets or traffic. This finding motivated a correlation analysis of the frequency power of all recordings per LUT that effectively unravelled site-specific frequency correlations. The proposed method enabled us to reveal characteristic frequency communities, which reflected individual mixtures of sound sources that differed for the examined LUT. Our land use mixes, characterised by different gradients of built-up and green areas, are well resembled by these findings, as areas with high anthropogenic influences exhibit distinct patterns from natural areas. For areas with high anthropogenic influence, frequencies < 100 Hz correlate highly with frequencies up to 9 kHz and frequencies > 4 kHz correlate strongly with all frequencies above. Contrary, in green areas, two ‘large’ rectangular communities emerge below and above 9.5 kHz. In addition, the recording stations with both urban and natural surroundings showed properties from both, e.g., correlations between >100 Hz up to 9 kHz or distinct frequency communities, but with a varying lower frequency bound for the community below 9.5 kHz. The observed frequency communities and the associated modality of the R^2^-distribution consequently yielded valuable information on the complexity of different urban AEs. Our results give evidence that correlations between frequency powers are a promising approach to describe the urban acoustic environment, as they not only map the frequency spectrum, but also consider the temporal dimension at the same time.

In this work, we chose to correlate all recordings per LUT, but there were virtually no limits for grouping recordings (e.g., by daytime, season, weather conditions, etc.) to reveal specific frequency communities for the groups of interest. Additionally, an advantage of this method is that frequency power is normalised in correlation analysis. Many ecoacoustic indices rely on the absolute power of the signal (e.g., LAeq, BIO, M), but as the measured power depends highly on the distance of the microphone to the sound source, measurements relying on absolute power vary heavily. By normalising frequency power, microphone positioning should have a much smaller influence on the analysis outcome. Another advantage of this method is that the dimension of the outcome (i.e., the correlation matrix) is only dependent on the number of frequency bins chosen. Therefore, follow-up analyses (e.g., image classification and network analysis) of the FCMs circumvent the problem of differing dimensions of the input, which can be a problem, e.g., in machine learning [53]. In addition, this property of FCMs represents a possible solution to the problem of the visualisation of large longitudinal audio datasets [31,54], as their dimension is only dependent on the number of frequency bins.

Besides these advantages, our analyses have several limitations. Although we analysed over 390,000 min of recordings in nine different LUTs, this still only represents a part of the entire urban AE. While our results seem plausible and robust commonalities are identified between all LUTs, they are not representative for the entire urban AE. In addition, because of the COVID-19 lockdown measures, we were not able to analyse an entire year of frequency dynamics in the urban AE. This may have helped with the explanation of some unexplained frequency communities found in different LUTs for different times. These unexplained patterns are not necessarily a limitation of our analyses, but a research gap whose solution could help in explaining frequency dynamics more vividly. Another limitation that needs to be stressed is that power spectra were constructed using 3-min recordings, possibly masking short-time acoustic events occurring at time scales below this sampling resolution.

In future works, our results can be adapted in many ways. For instance, we showed that 1024 frequency bins were sufficient to describe the urban AE on a linear scale. Median Power Spectra revealed that the highest variance in the urban AE was contained in the frequency band up to 9 kHz and most power up to 13 kHz. Therefore, future audio recordings could be restricted to a sampling frequency of 18 or 26 kHz to save energy and storage space. Power spectra also revealed higher variance between frequency bins up to 4.5 kHz. Thus, a logarithmic scale (e.g., Mel scale) might extract information of the urban AE more precisely. Although Bradfer-Lawrence et al. [26] advise recording periods of 120 h to represent the AE of one site, this may not be feasible for the urban AE. For instance, information about biophonic activity is highly dependent on season and should be considered when setting the aims of an urban AE study.

Furthermore, explicitly including the interplay between distinct frequency bands in model development of the urban soundscape could add another layer of complexity to these approaches. Current model limitations, e.g., use of loudness-based or psychoacoustic indicators as well as limited validation due to lack of extensive datasets [24], could benefit from AE analyses that consider the full available time–frequency information, e.g., by using FCMs like those that have been put forward in this work. The same applies to user-based approaches to evaluate the perception of the AE. FCMs quantify the AE and can be connected to perceptual indices to reveal how frequency dynamics are associated with perceived acoustic quality. The uniform visualisation for differing time scales could lead to an intuitive understanding of acoustic quality and its interlinked frequency dynamics.

## 6. Conclusions

In this work, we analysed one of the most comprehensive spatially and temporally high-resolution datasets of the urban AE to date. We analysed 271 days of audio recordings and found communalities as well as distinctions between LUTs and time periods. Normalised Spectrograms revealed time- and LUT-dependent frequency dynamics and uncovered frequency patterns that were otherwise hidden. In this regard, our results can be adapted in future studies of the urban AE to increase precision and efficiency of audio sampling and/or adjust acoustic indices. Lastly, we demonstrated a novel approach using FCMs to aggregate the information of the time–frequency domain of a large longitudinal audio dataset efficiently. We found that FCMs reflect the information of frequency communities and are a promising approach to describe or classify the urban acoustic environment. The symmetry and time-independent dimensions of FCMs hold great potential for efficient AE visualisation and further analyses such as machine learning or network analysis.

We found that frequency dynamics provide valuable information about the AE at specific locations. Considering them will add value to different domains such as perceptual soundscape research or psychoacoustics and will consequently help to broaden our understanding of the impact of the AE on human health.

## Data Availability

The data presented in this study are available on request from the corresponding author. The data are not publicly available due to potential privacy issues.
